# Tannic acid inhibits lipid metabolism and induce ROS in prostate cancer cells

**DOI:** 10.1038/s41598-020-57932-9

**Published:** 2020-01-22

**Authors:** Prashanth K. B. Nagesh, Pallabita Chowdhury, Elham Hatami, Shashi Jain, Nirnoy Dan, Vivek Kumar Kashyap, Subhash C. Chauhan, Meena Jaggi, Murali M. Yallapu

**Affiliations:** 1 Department of Microbiology and Immunology, School of Medicine, University of Texas Rio Grande Valley, McAllen, TX 78504 USA; 2 Department of Pharmaceutical Sciences and Center for Cancer Research, University of Tennessee Health Science Center, Memphis, TN 38163 USA; 3 Tumor Initiation and Maintenance, Sanford-Burnham Medical Research Institute, La Jolla, California 92037 USA; 4 Department of Pathology, Moores UCSD Cancer Center, and Sanford Consortium for Regenerative Medicine, University of California, San Diego, La Jolla, CA 92037 USA

**Keywords:** Cancer metabolism, Pharmaceutics

## Abstract

Prostate cancer (PCa) cells exploit the aberrant lipid signaling and metabolism as their survival advantage. Also, intracellular storage lipids act as fuel for the PCa proliferation. However, few studies were available that addressed the topic of targeting lipid metabolism in PCa. Here, we assessed the tannic acid (TA) lipid-targeting ability and its capability to induce endoplasmic reticulum (ER) stress by reactive oxygen species (ROS) in PCa cells. TA exhibited dual effects by inhibiting lipogenic signaling and suppression of lipid metabolic pathways. The expression of proteins responsible for lipogenesis was down regulated. The membrane permeability and functionality of PCa were severely affected and caused nuclear disorganization during drug exposure. Finally, these consolidated events shifted the cell’s survival balance towards apoptosis. These results suggest that TA distinctly interferes with the lipid signaling and metabolism of PCa cells.

## Introduction

Prostate cancer (PCa) is a leading disease with a majority of incident cases and stands next to lung cancer in mortality rates among men populations^1^. The poor prognosis for PCa patients accounts for higher mortality rates with 5 year survival rate in metastatic cases was less than 35%^2^. Several signaling players and growth factors contribute towards the cell growth of prostate cancer^3^. However, the identification of key molecular targets is the prime motive of precision medicine in clinic^4^. Reprograming of onco-metabolism is an established cancer hallmark and have attained clinical significance as therapeutic molecular target in PCa^5^. The functional imbalance between oncogenic and tumor suppressive genes, and poor oxygen with meager nutrient availability in tumor microenvironment (TME) drives the changes in metabolic phenotype of the cancer cell^6^.

Extremely proliferative cancer cells have a powerful lipid and cholesterol avidity that they fulfill either by enhancing the absorption of exogenous (or nutritional) lipids and lipoproteins or by overactivation of their endogenous lipogenesis and cholesterol synthesis^6^. Genetics of lipid profiling analysis of prostate cancer showed characteristic dysregulation of lipid metabolism that promotes invasiveness and progression of the disease^7^. The imbalance in the lipid metabolism of cancer cells is predominantly due to aberrant de novo lipogenesis, induced biosynthesis of steroid hormones, and fatty acid oxidation that endorses the tumor proliferation^8^. Alterations of lipid metabolism in prostate cancer patients was evidenced through *in situ* studies^9^. The etiology behind lipid metabolism aberrations in PCa was due to the overexpression of lipogenic and peroxisomal enzymes like fatty acid synthase (FASN)^10^ and α-methylacyl-CoA racemase (AMACR)^11^, respectively. It is also well known that altered lipid metabolism is a prime hallmark of cancer^12^. Thus, therapeutic targeting of dysregulated metabolic lipid signaling might be an innovative strategy for promising therapies against prostate cancer.

The endoplasmic reticulum (ER) is a vital organelle that serves as the site for biosynthesis of proteins and its post translational modifications in the cell. Intracellular abnormalities related to the function of ER, such as activation of unfolded protein response (UPR), trigger ER stress^13^. Prevalence of ER stress is also associated with the oxidative stress in cancer cells^14^. Oxidative stress causes cellular damage by ROS production in cells with compromised antioxidant resistance mechanism. Induction of ROS causes redox imbalance that aggravates ER stress signaling by diminishing the competence of protein-folding mechanisms, resulting the rise of unfolded protein levels^15^. These correlations between ER stress and ROS mechanisms can be implicated in therapeutic targeting in cancer cells.

Maintenance of intracellular ROS homeostasis is necessary for normal cell proliferation and survival^16^. However, excessive accumulations of ROS triggers oxidative damage and causes imbalances in the redox status of the cell^17^. The ROS accumulations were due to the declination of ROS scavenging abilities like reduction of enzymatic activity such as superoxide dismutase and glutathione peroxidase^18^. This induced ROS considerably affects the liveliness of membrane bilayers and disrupts their integrity. Hydroxyl radical (HO**˙**) and hydroperoxyl (HO**˙**_2_) are the most prevalent ROS species that affect the lipids^19^. Succinctly, these reactive compounds can also affect the permeability and fluidity of membranes consisting of lipid bilayers that remarkably affect cell survival and integrity^20^.

Tannins are a class of polyphenolic compounds derived from plant origins especially found in fruits, red wine, coffee, nuts, and beans^21^. Tannic acid (TA) is a prominent member of tannins family and is comprised of gallic acid molecules esterified to several functional hydroxyl groups^22^. TA exhibits potential anticancer activity against several cancer cell lines^23–25^. In our previous study, we demonstrated the mechanistic anticancer role of TA in prostate cancer. From these results, we found that TA induced ER stress through UPR, subsequently promoting apoptosis. However, the correlation between ER stress induction and apoptosis signaling in our previous study was not examined  fully^26^. Thus, in this study, we evaluated TA’s ability in ROS induction and its ability to interfere lipid metabolism as well as disruption of membranes which subsequently destabilizes PCa cellular integrity.

## Results

### Dose dependent anti-proliferative effects of TA

We validated TA’s anti-proliferative activity against prostate cancer cells (C4-2 and PC-3) through the xCELLigence system. After treatment, we observed the dose dependent inhibitory pattern of TA during 10 and 20 µM concentration in both cell lines **(**Supplementary Fig. 1A**)**. Through xCELLigence proliferation studies we affirmed the inhibitory effects of TA on C4-2 and PC-3 cells. Similar growth inhibitory patterns were observed in invasion and migration studies of xCELLigence system during TA treatment of C4-2 and PC-3 cells **(**Supplementary Fig. 1B,C**)**.

**Figure 1:**
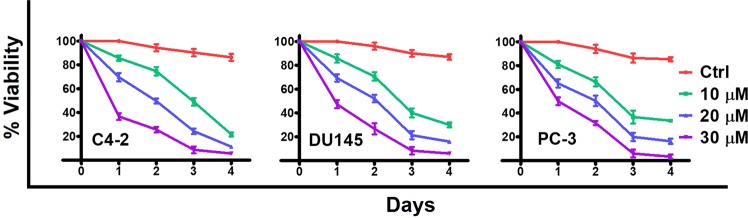
*In vitro* cytotoxic efficacy of TA on prostate cancer cells. (**A)** Kinetic profile of proliferation by PCa (C4-2, DU145 and PC-3) cells after treatment with 10, 20, and 30 µM TA was evaluated by Countess Automated Cell Counter, determining the percentage of cell viability from 0 to 4 days. Data presented as mean ± standard error of the mean (n = 3).

The pharmacological  effects of TA were demonstrated in C4-2, DU145, and PC-3 cell lines through kinetic studies by trypan blue dye exclusion method. The treatment of TA with three different concentrations of 10, 20, and 30 µM was performed for 4 consecutive days. We observed a progressive decrease in percent cell viabilities of treated cells with the extended exposure till 4 days. Additionally, we observed a characteristic growth inhibition of cells during high dose (30 µM) drug exposure in all PCa cells **(**Fig. 1**)**. Since 30 µM of TA induced significant cell death, we have chosen 10 and 20 µM of TA for subsequent studies. Altogether, we observed dose dependent effects of TA against C4-2, DU145, and PC-3 cells.

### TA triggers ER stress signaling in PCa cells

**Figure 2:**
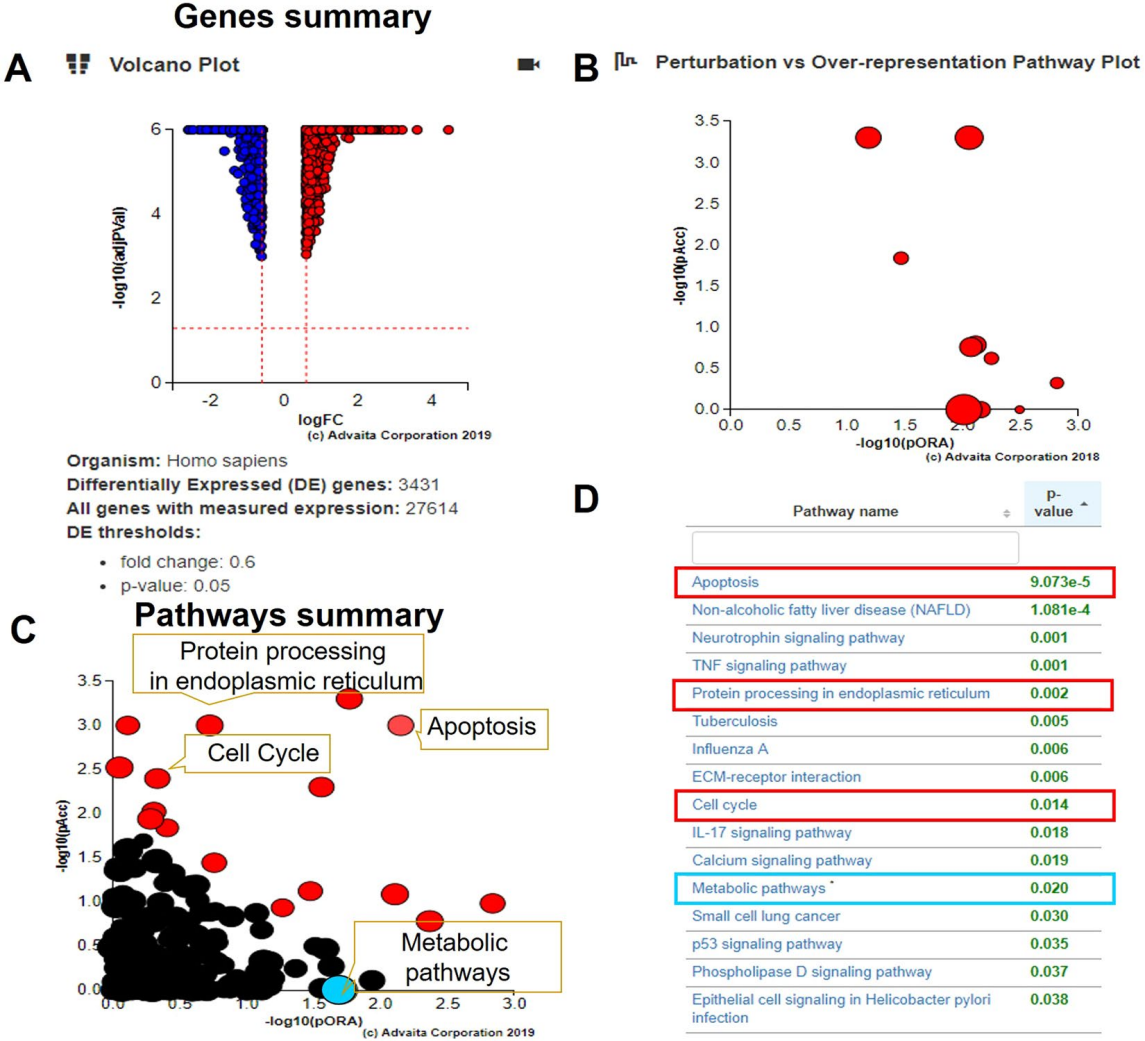
Gene regulatory effects of TA in PCa cells through ipathwayGuide analysis. (**A**–**D**) ipathwayGuide analysis showing effect of TA on prostate cancer cells. (**A)** Volcano plot of 3431 differentially expressed genes (DE) (**B**) Perturbation vs over-representation pathway plot. (**C)** Pathway summary **D)** The major affected signaling pathways after treatment of TA on PCa cells.

**Figure 3:**
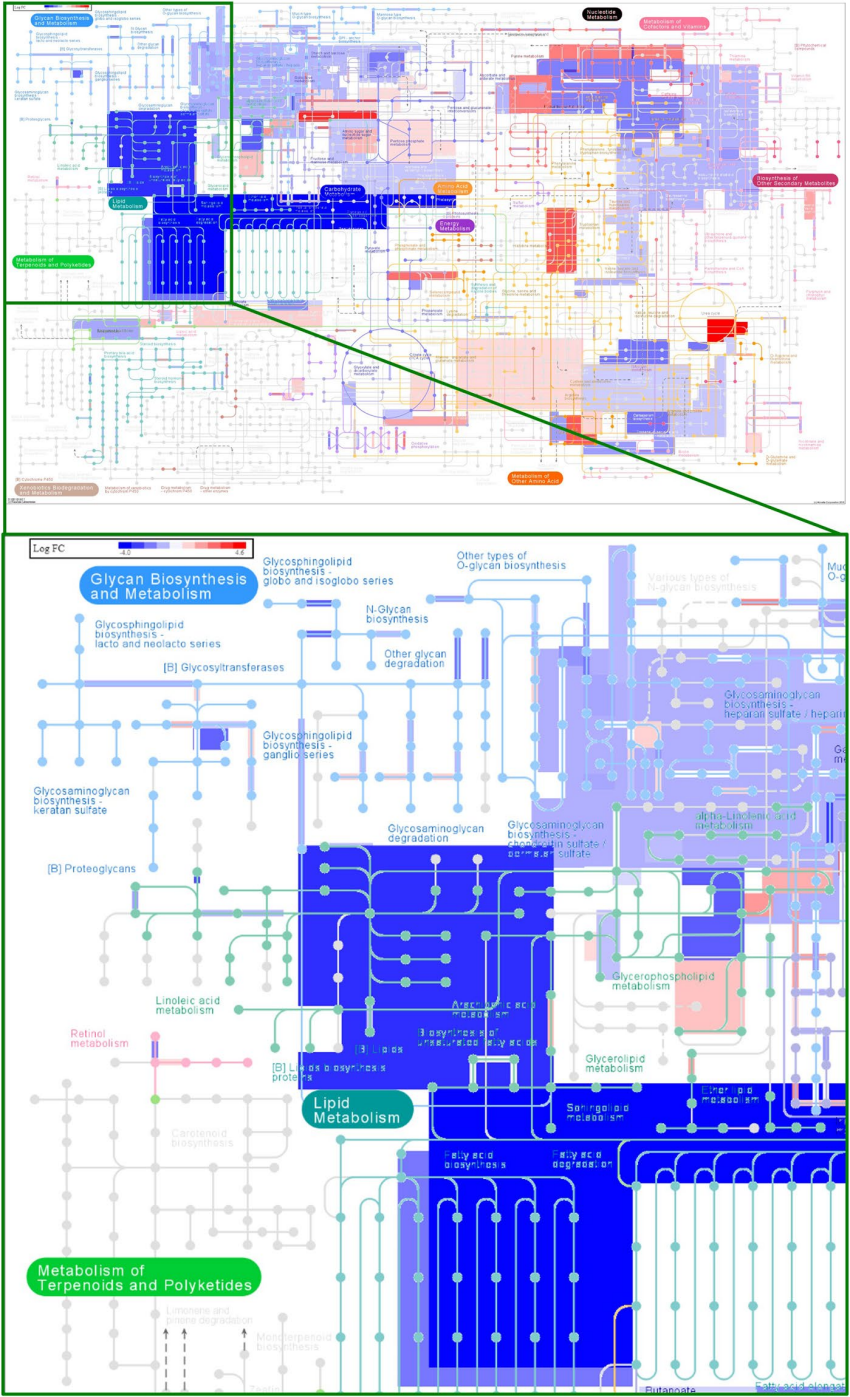
Tannic acid deregulates lipid metabolism by ipathwayGuide analysis. The whole landscape of lipogenesis pathway that was inhibited by TA treatment on prostate cancer cells as determined by iPathwayGuide analysis. (Induced-represented in red color; Inhibited-represented in blue color).

In our previous study, we demonstrated the molecular regulation of TA in inducing ER stress signaling of PCa cells resulting in apoptotic induction^26^. Further, we substantiated that TA specifically induces ER stress by UPR mechanism in prostate tumor cells by micro array study through ipathwayGuide analysis **(**Fig. 2A–D**)**. The ipathwayGuide analysis enables to understand mechanistic effects of TA on numerous cellular growth and regulatory pathways. The results of ipathwayGuide analysis showed TA has influential effects on the regulation of genes related to protein processing in ER **(**Fig. 2D**)**, besides the activation of proapoptotic signaling. Amongst the most affected pathways we observed apoptosis, protein processing in ER, cell cycle and metabolic pathways are closely related to oncogenesis. In our previous results^26^, we elaborately disclosed about the molecular effects of TA on apoptotic, protein processing in ER (UPR) and cell cycle mechanisms. However, TA’s effect on metabolic pathways and its relation towards ER stress have been not explored. The metabolic dysregulation in cells caused by TA was visualized through landscape of metabolic pathways **(**Fig. 3**)**. From the panoramic metabolic landscape, we evidently observed the down regulation of lipid metabolism related gene (represented as blue color) while there was no distinct inhibition of glucose and amino acid metabolism. These results annotated the specificity of TA in inhibiting lipid metabolism. In this view, our following studies were focused on TA’s inhibitory role on fatty and lipid metabolism. Altogether, we affirmed that TA strictly affects ER stress signaling and protein processing in prostate cancer cells this in turn directly or indirectly effects the biogenesis and metabolism of lipids.

### PCa cells produce ROS following TA treatment

**Figure 4:**
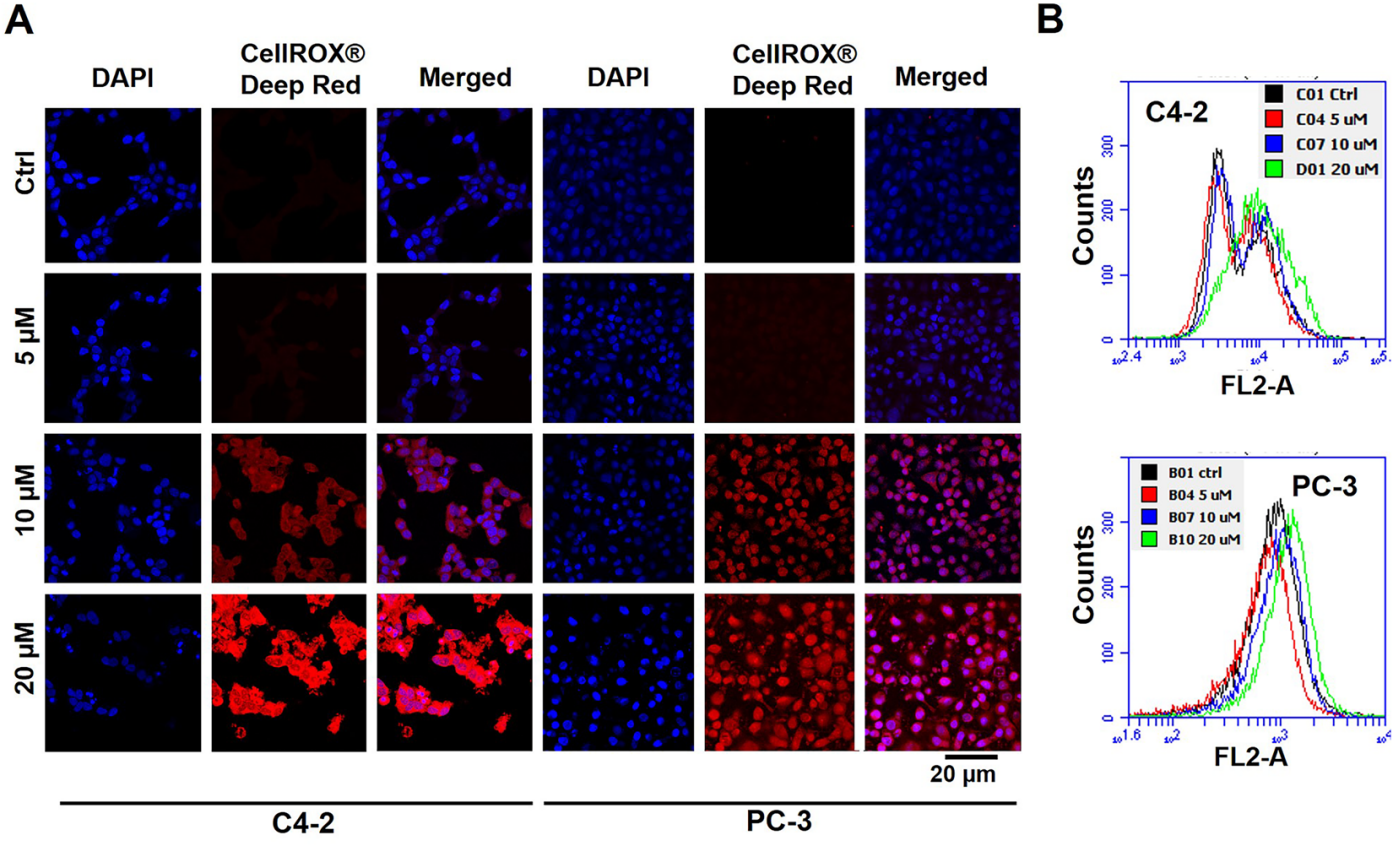
Tannic acid induce intracellular reactive oxygen species on prostate cancer cells (**A**) Immunofluorescence assay determining treatment with 10 to 20 µM TA induces ROS production in C4-2 and PC-3 cells as determined by CellROX Deep Red oxidative stress reagent (5 μM; Life Technologies) (**B**) Quantitative analysis of ROS production was further validated using C4-2 and PC-3 cells by flow cytometry, showing a decrease in mitochondrial membrane potential, thus eventually leading to cell death. Data presented as representative from triplicates  .

Previous reports have stated that polyphenols exhibit oxidative stress in various cancer cells and tumor models^27–29^. Since TA is a polyphenol, we assessed its oxidative stress ability through ROS measurement. Cells (C4-2 and PC-3) were exposed to TA for 24 h and exposed to CellROX for 30 min. We observed an incremental increase of fluorescence intensity in treatment groups from 10 to 20 µM concentration **(**Fig. 4**)**. From the microscopic studies, at 40X magnification we observed that cells exhibited red fluorescence after the TA’s exposure (10 µM), and this fluorescence has been enhanced during 20 µM treatment **(**Fig. 4A**)**. Additionally, from the flow cytometric results, we observed a prominent right shift of peak in the samples treated with TA in comparison to untreated controls, annotating the fluorescence increase in the cells **(**Fig. 4B**)**. The rise in the intracellular fluorescence is directly proportional to the amount of oxidative stress, thus elucidating the ROS induction by TA. The progressive shift of peaks was consistent with the dose dependent increase of drug treatment in the flow cytometric studies. These qualitative results of ROS studies were affirmed through confocal studies, while the quantitative results were validated through flow cytometric studies. Also, these outcomes indicate that TA may possibly have a role in decreasing mitochondrial membrane potential, thus driving cell survival status toward apoptosis.

### Measurement of lipid content in PCa cells

**Figure 5:**
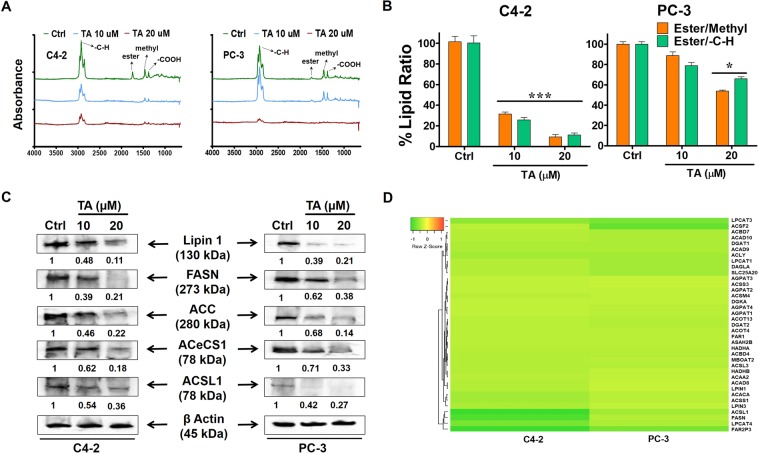
Tannic acid interferes with Lipid biogenesis and metabolism in prostate cancer cells. (**A)** FTIR spectral analysis of lipid content as extracted from C4-2 and PC-3 cells after TA treatment by Bligh and Dyer protocol. (**B)** The percent lipid ratios (ester/methyl and ester/-C-H) of cellular lipid extracts in C4-2 and PC-3 cells. Values obtained from the FTIR measurements (n = 6). **(C)** Western blot analysis showing dose dependent TA treatments affects the gene expression related to lipogenic signaling pathway as demonstrated by lipogenic proteins such as Lipin 1, Fatty acid synthase (FASN), Acetyl-CoA carboxylase (ACC), Cytoplasmic acetyl-CoA synthetase (ACeCS1) and Mammalian long-chain acyl-CoA synthetase (ACSL1). The protein ladder used for these bolts’ ranges from 10 kDa to 250 kDa (Bio-rad cat#1610374, Hercules, CA). All full-length blots are presented in Supplementary Information. (**D)** Heat map analysis of lipid metabolism related genes during TA treatments on C4-2 and PC-3 cells. Data presented as mean ± standard error of the mean (n = 3). * and *** Represent level of significance with P (<0.05 and 0.001) with respect to control.

Previous reports have revealed that the measurement of lipid profiles was evaluated through FTIR spectroscopy^30^ and lipid staining procedures^31^. Since TA has anti-proliferative potential on PCa cells^26^, it is imperative that its influence on lipid biogenesis and metabolism needs to be understood. In this view, we extracted lipids from the C4-2 and PC-3 cells after treatment by TA by Bligh and Dyer protocol as explained in the methods section. To demonstrate the percent of lipid content in treated cells, we assessed these samples through FTIR analysis. The fraction containing lipids was collected from the TA-treated cells from the procedure mentioned in methods section. When the lipid samples were measured for the absorbance on the FTIR, we observed the peaks were curtailed during the TA treatments **(**Fig. 5A**)**. Interestingly, the absorbance of peaks was diminished in a dose dependent manner with 10 and 20 µM treatments in both PCa cells. The decrease in the peak absorbance indicates annotating the reduction of lipids in the cells’ samples, which may be due to the therapeutic effects of TA.

Further, the percent ratios of ester groups (peak at 1746 cm^−1^) with the methyl groups (peak at 1450 and 1746 cm^−1^) were measured to demonstrate the proportion of fatty acid and lipid content of cells after TA treatments. We observed dose dependent decrease of percent lipid ratios in PCa (C4-2 and PC-3) cells. Additionally, we also measured the percent ratios of ester groups (peak at 1746 cm^−1^) with the -C-H groups (peak at 2929 cm^−1^) **(**Fig. 5B**)**. There was dose dependent reduction of percent ratios in both PCa cells with similar results as mentioned above. Also, the peak at 1395 cm^−1^ was diminished during TA treatments in both cells refers to -COOH groups. The ester, methyl, -C-H, -COOH are the prime molecular components of fatty acid and lipids. The decrease in the proportion of the mentioned groups clearly denotes the reduction of intracellular fatty acid and lipids during TA treatments. From these studies, we can clearly state that cellular lipid profiles during TA treatment were drastically affected and reduced.

### TA averts tumor lipogenesis in prostate cancer cells

Cancer cells with induced lipogenesis play a preeminent role in oncogenesis^32^ and chemoresistance^33^. To demonstrate the TA’s influence on cancer cells’ lipid metabolism, C4-2 and PC-3 cells were exposed to TA in dose dependent manner and a western blotting study was performed. As depicted in Fig. 5C, the protein profiles in the prostate cancer cells were reduced with TA’s intervention. The lipogenesis or fatty acid synthesis regulation was affected due to the variation in gene expression that modulates the expression of proteins related to lipogenic signaling^34^. Henceforth, we assessed TA’s activity in reduction of lipid droplets at intracellular level, which was regulated by lipogenic proteins such as Lipin 1, Fatty acid synthase (FASN), Acetyl-CoA carboxylase (ACC), Cytoplasmic acetyl-CoA synthetase (ACeCS1), and Mammalian long-chain acyl-CoA synthetase (ACSL1). All full-length blots were provided in the supplementary information. Here, the expression of the proteins were found to be reduced in both 10 and 20 µM of TA treatments. Also, we evaluated the gene expression profiles of proteins related lipid metabolism during TA treatments on both C4-2 and PC-3 cells **(**Fig. 5D**)** through micro array analysis. These results were coherent and coinciding with the results obtained through *in vitro* and western blot studies. Altogether, these results suggest TA’s mechanistic role in inhibiting lipogenic signaling, thus correlating with our results discussed in the above section.

### TA interferes with plasma membrane integrity and nuclear organization

**Figure 6:**
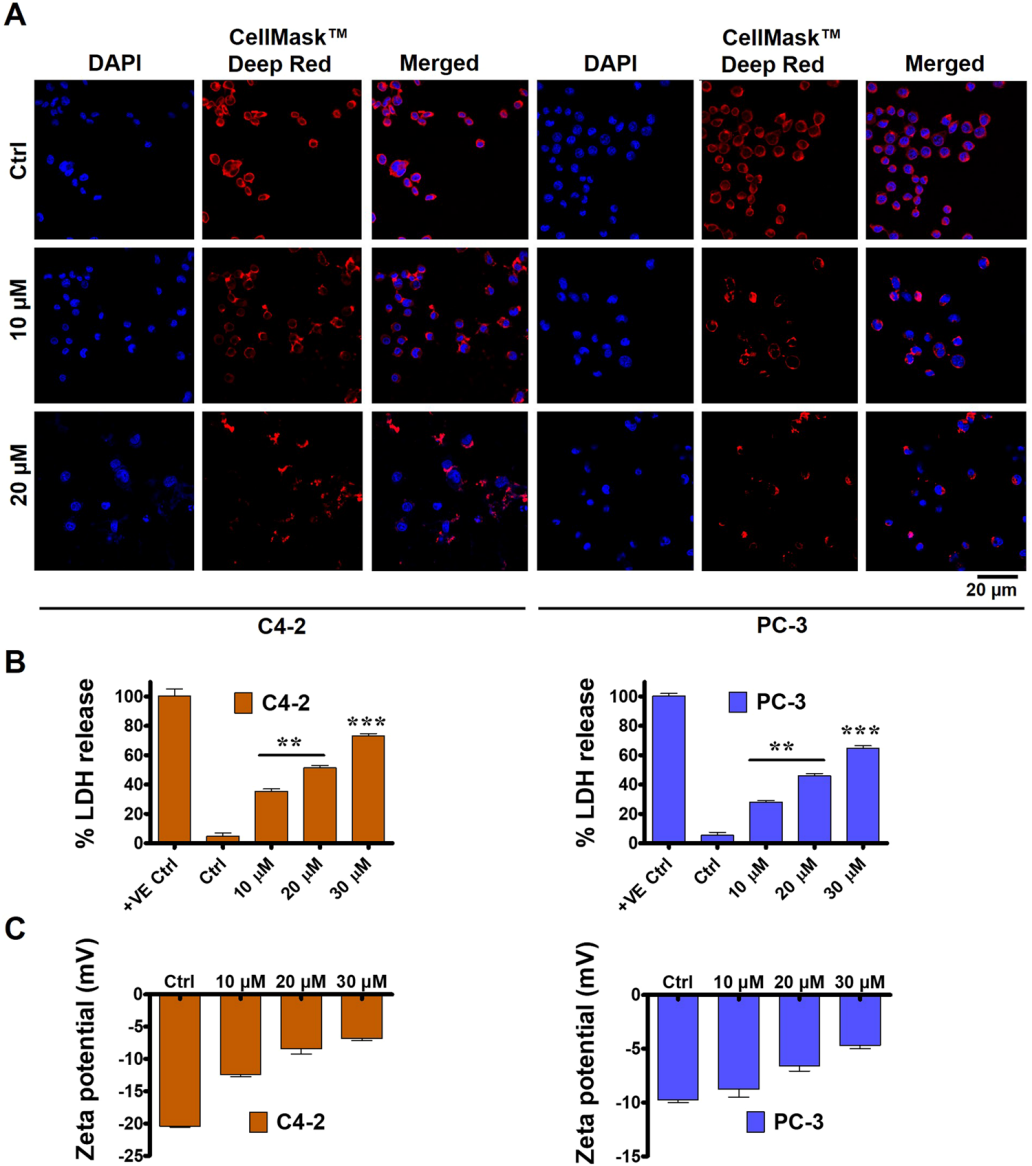
Effect on plasma membrane integrity on prostate cancer cells with TA treatment were determined. (**A)** Immunofluorescence showing plasma membrane of PCa (C4-2 and PC-3) cells were severely affected in a dose dependent fashion with TA treatment as shown by plasma membrane stain, CellMask Deep Red. (**B)** Lactate dehydrogenase (LDH) levels as measured using CytoTox-ONE Homogenous Membrane Integrity Assay kit (Promega) were used to study the effect of TA on PCa membrane, demonstrating levels for damaged membrane with TA exposure and eventually leading to PCa cell death. (**C)** Zeta potential measurements were used to assess the surface charge of PCa cells after 10, 20, and 30 µM TA treatment, showing that TA induces cell death by neutralizing the negatively charged functional membrane. Data presented as mean ± standard error of the mean (n = 3). ** and *** represent level of significance with P (<0.01 and 0.001) with respect to control.

We evaluated the plasma membrane integrity through confocal studies by staining the plasma membrane with CellMask Deep Red plasma membrane stain. The drug treatments showed profound membrane disorganization in both PCa (C4-2 and PC-3) cells. The confocal images of cells show the fluorescence intensity was feeble and reduced during TA treatments in dose dependent fashion. The control cells exhibited uniform round-shaped red fluorescence staining intact plasma membrane, while the extent of roundedness decreased and displayed nonuniformity during 10 µM treatments. Further, this extent of uniformness was dwindled and distinct during 20 µM in both cells **(**Fig. 6A**)**. This decline in the fluorescence roundness of cells indicates that the structure of the plasma membrane was not healthy and normal, showing TA’s molecular specificity towards the lipid bilayer.

Further, to prove that the plasma membrane is not intact and was damaged, we evaluated the release of LDH, a marker for the damaged membrane^35^. The percent of LDH release during the 20 µM treatment has increased to around 50% and 40% percent in both C4-2 and PC-3 PCa cells, respectively, in comparison to untreated controls **(**Fig. 6B**)**. In this study, we employed 9% (W/V) of triton X-100 in water as a positive control with 100% LDH release. These results annotate that the plasma membrane was not functional, affecting its selective permeability attribute, resulting in cell death due to TA exposure.

Further, we assessed the surface charge of the treated cells to validate the functionality of the membrane through Zeta potential measurements. The results of Zeta potential studies have revealed that the surface charge of the treated cells exhibited more positive values, i.e., less negative, than untreated cells. The surface charge of the cells treated with the drug at 10 and 20 µM concentrations was found to be −12.4 and −8.43 mV, respectively, in C4-2 cells, while the control cells exhibited a surface charge of −20.4 mV **(**Fig. 6C**)**. In PC-3 cells, the surface charges were found to be −8.76 and −6.57 during 10 and 20 µM treatments, respectively, which were less negative than control cells (−9.77 mV). In total, these results conclude that TA disrupts the plasma membrane functionality, which might be causative for the induction of cellular death.

**Figure 7:**
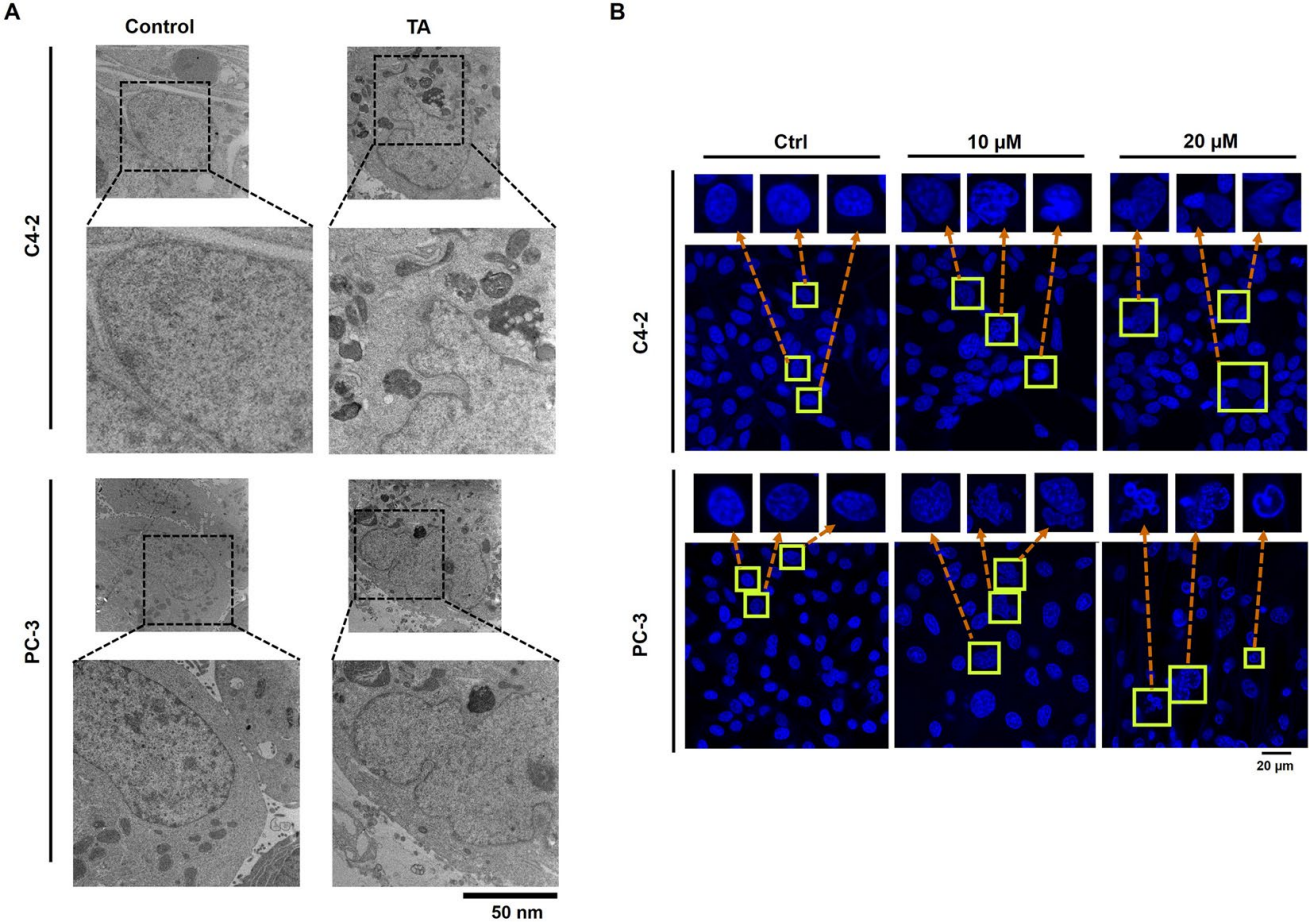
Nuclear organization on treatment with TA on prostate cancer cells. (**A)** TEM images demonstrating destabilized nuclear integrity of C4-2 and PC-3 cells (**B)** DAPI staining showing clear disorganization of nuclear morphology with TA exposure as shown by the confocal images.

The nuclear envelope is also composed of lipid bilayers along with numerous accessory proteins that provide a physical barrier between genetic material and cytoplasm^36^. Disruption of the nuclear envelope in the cell with constitutive gene expression exposes chromosomal DNA to cytoplasmic milieu, triggering DNA damage that drives the cell towards apoptosis^37^. To validate the effects of TA on nuclear lipid bilayer, we studied the changes that would occur in the nuclear morphology. To validate the nuclear changes, we performed TEM imaging of C4-2 and PC-3 cells exposed to 10 µM of the drug. TEM images of the TA-treated cells showed mutilated morphology of the nucleus that destabilized the nuclear integrity **(**Fig. 7A**)**. Further, to support our results of nuclear morphological changes during TA exposure, we performed the DAPI staining procedures. From these studies, we observed similar morphological changes in both cells **(**Fig. 7B**)**. The number of cells with distorted nuclear morphology was prominently observed during 10 and 20 µM of TA treatments. The mutilation of the nucleus might be due to the membrane disruption activity of TA on the nuclear envelope. In summary, these changes in the nuclear structure trigger the progression of apoptosis of prostate cancer cells during TA treatment.

### Chemo-sensitizing ability of tannic acid

**Figure 8:**
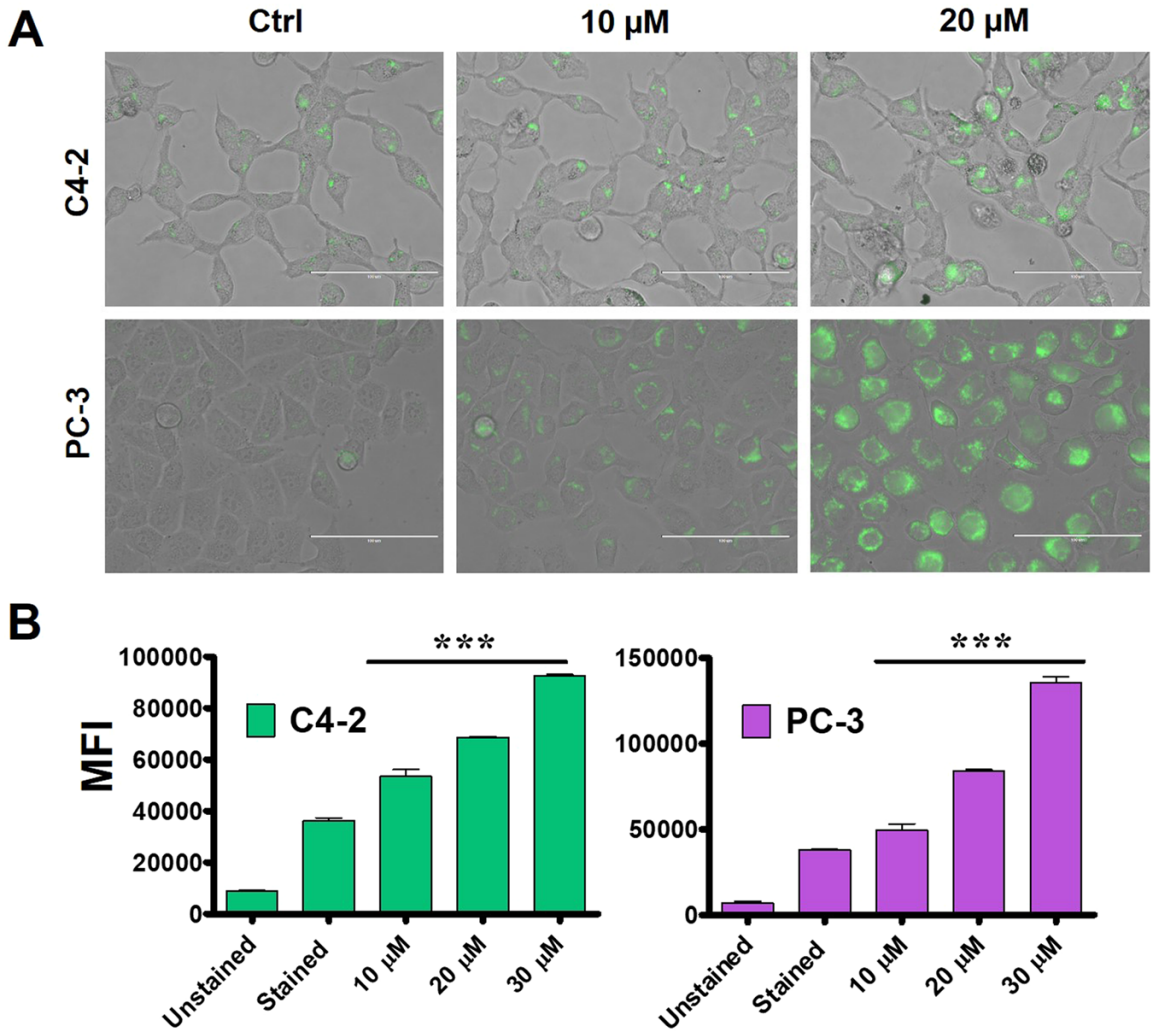
Chemo-sensitizing ability of TA. (**A,B)** Rh123 accumulation study (**A)** Qualitative depiction of Rh123 uptake by the PCa (C4-2 and PC-3) cells after exposure to 10 and 20 µM of TA for 24 h, showing enhanced accumulation with increased TA concentrations. (**B)** Quantitative analysis as assessed by flow cytometer, showing dye accumulation was in a dose dependent fashion with TA treatment in both the PCa cells. Data presented as mean ± standard error of the mean (n = 3). ***Represent level of significance with P (<0.001) with respect to control.

To assess the chemo-sensitive attributes of TA, we investigated the combinatorial implications of TA through Rhodamine 123 (Rh123) dye accumulation studies. The TA treated cells after 24 h were exposed to Rh123. The dye accumulations were evaluated using fluorescence microscopic and flow cytometric studies. From the fluorescence microscopic studies, we observed that the accumulation of dye was prominent during the TA treatments. Additionally, the fluorescence due to the accumulation of Rh123 was more profound during the 20 µM exposure than 10 µM of TA treatments **(**Fig. 8A**)**. Analogously, we demonstrated these results in quantitative manner through flow cytometric studies **(**Fig. 8B**)**. Results depicted showed increase of mean fluorescence intensity (MFI) in both C4-2 and PC-3 cells during the TA exposure in comparison with untreated and unstained controls. We observed a major shift of peaks towards the right side in the samples treated with 20 µM with respect to the untreated control, annotating the fluorescence accumulations of the dye.

**Figure 9:**
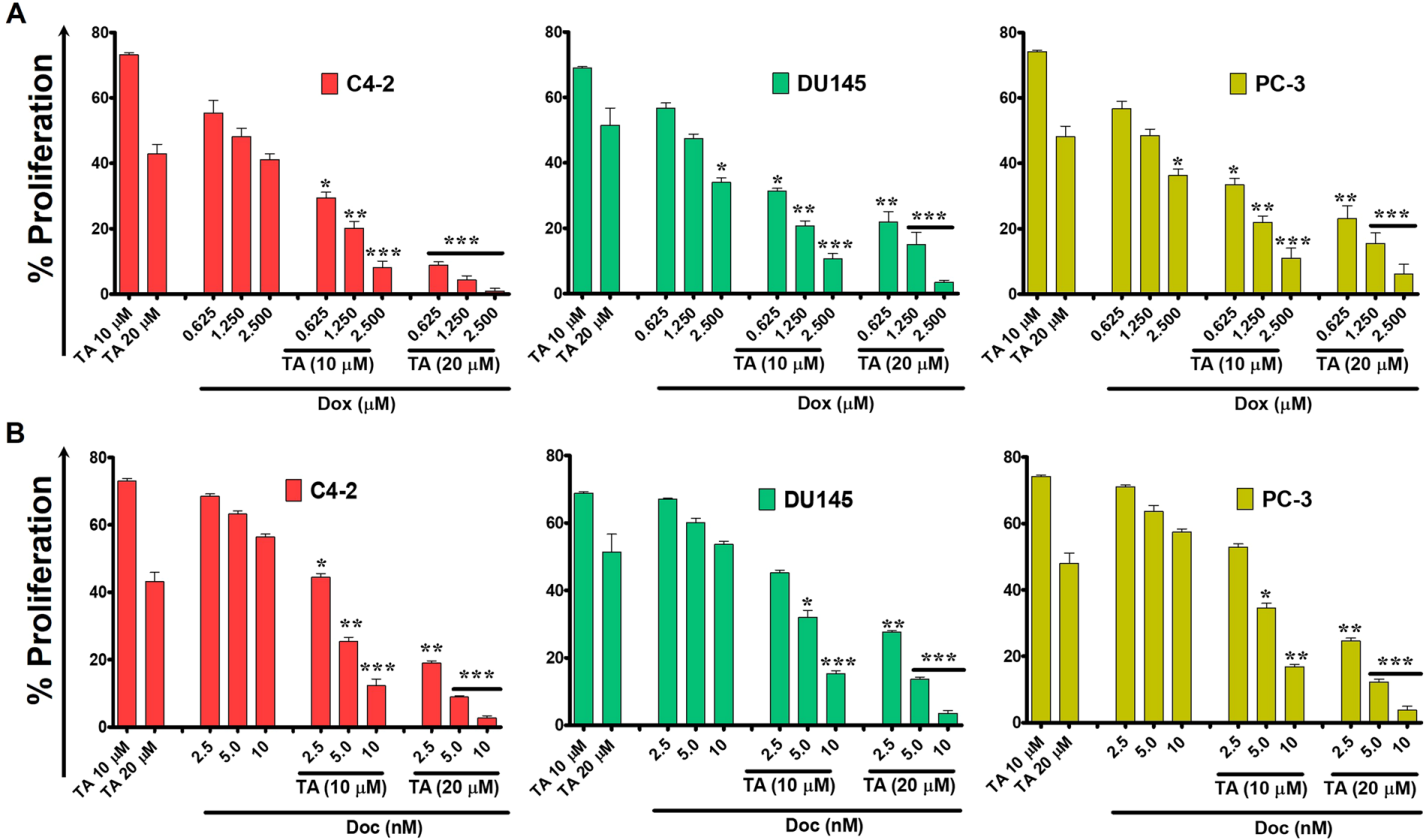
Tannic acid augments therapeutic efficacy of doxorubicin and docetaxel against prostate cancer cells. MTS proliferation assay was used to determine potential chemo-sensitization activity of TA in combination with FDA-approved chemotherapeutic agents (**A)** Doxorubicin and (**B)** Docetaxel demonstrating TA enhances the chemo-sensitivity ability of the drugs. Data presented as mean ± standard error of the mean (n = 3). *, **, and *** represent level of significance with P (<0.05, 0.01 and 0.001) with respect to control.

Here, we show dye accumulations are analogous to the drug accumulations to evaluate the chemo-sensitization concept of TA during standard Food and Drug Administration (FDA)-approved chemotherapies. To validate this phenomenon, we have treated the cells using varying concentrations of doxorubicin (Dox): 0.625, 1.25 and 2.5 µM in the presence of TA (10 and 20 µM). Also, the cells were exposed to varying concentrations of docetaxel (Doc): 2.5, 5 and 10 nM in presence of TA. The percent proliferations in both the C4-2 and PC-3 cell lines were reduced drastically during Dox + 20 TA µM and Doc + 20 TA µM exposure with respect to only Dox or Doc treatments **(**Fig. 9A,B**)**. With these results, we affirm the chemo-sensitization activity of TA in C4-2 and PC-3 cells.

**Figure 10:**
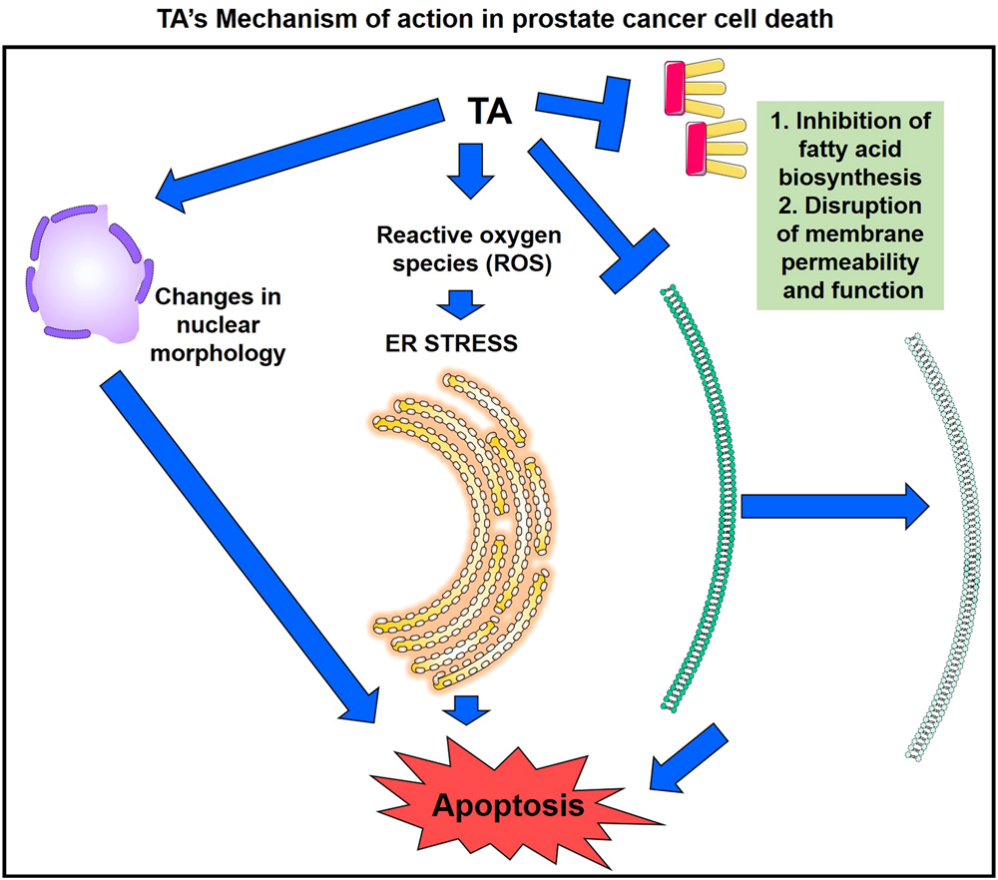
Schematic representation of mechanism of action caused by TA for leading to apoptosis in prostate cancer cells.

Overall from all these results, it was clear that TA induced intracellular ROS levels, causing the activation of ER stress. The induction of ER stress by TA, directly or indirectly interferes with lipid metabolism of PCa cell subsequently leading to the activation of apoptosis. Concisely, TA also played a pivotal role in the disruption of lipid signaling/metabolism and affected membrane functionality as well as disorganization of the nucleus **(**Fig. 10**)**. Comprehensively, these events triggered apoptosis in prostate cancer cells during TA therapy.

## Discussion

Chemotherapy remains a mainstay pillar in cancer therapies. Targeting logarithmic proliferating cancer cells by chemotherapy produced expectable clinical outcomes and extended survival rates^38^. Advances in chemotherapies since the last decade have revamped the therapeutic management of prostate cancer^39^. Among these therapeutic regimens, chemotherapy remains the mainstay treatment choice against various cancer subtypes^40^. However, in order to administer accurate, personalized medicine for specific cancer subtypes, it is essential to identify new molecular targets in the form of mutations^41^. Among the newly identified targets, disabling anti-oxidant activities by chemotherapeutic drugs was the most reliable approach that subsequently promoted ROS induction, leading to cell death^42^.

Phytochemicals are plant-derived compounds exhibiting chemo-preventive activity against various cancers^43,44^. Besides their chemo-preventive roles in cancer, phytochemicals also induce ROS production by triggering the imbalances in the intracellular redox status, leading to oxidative damage-based cell death^45,46^. Similarly, in the current study, we found observations of ROS induction in PCa cells during TA treatment. These results were validated through confocal studies of CellROX staining and showed ROS induction during dose dependent treatments, with our flow cytometric results subsequently substantiating our observations. The events after ROS induction at the cellular level included DNA damage that resulted in genomic instability, triggering proteolytic degradation and instigating signaling kinases^47^.

At the intracellular level, induction of ROS promotes ER stress mechanism leading to apoptosis and further ROS interferes with membrane lipids thereby altering the membrane permeability^48–51^. The induction of ER stress affects the homeostasis of the cellular lipid metabolism^52^. Previous studies have shown to deregulate the lipid metabolism by ER stress mechanism in cancer cells during treatments^53^. Similarly, in our current project, the ROS induced by TA treatments promoted the ER stress induction in both C4-2 and PC-3 cells. To ensure the inhibition of lipids, we performed lipid membrane staining procedures through which we asserted that TA exposure would have encouraged the deregulation of lipid biogenesis and metabolism. Since the cancer cell membranes are composed of lipid bilayers^54^, we performed fluorescence membrane staining with CellMask dye for the cells to ensure the plasma membrane integrity during drug treatments. Interestingly, we observed that the intactness and functionality of the plasma membrane was deteriorated, causing imbalances in the cell survival mechanisms. Additionally, these observations were substantiated through the results of Zeta potential and membrane integrity studies. Further, TA also interferes with the signaling related to lipid biogenesis of the C4-2 and PC-3 cells, which was affirmed by western blot results and iPathwayGuide analysis. At a molecular level, TA treatments inhibited the FASN, a key protein of lipogenesis of neoplastic cells^55^ that alters the intracellular lipid content, which is a prerequisite for a cell to undergo mitosis^56^. Further, these results were supported by FTIR studies. Altogether, TA exhibited bipolar effects against lipid metabolism with inhibition of lipogenesis on the other side; it also triggered the disruption within membrane integrity and functions such as affecting permeability resulting the release of LDH, which is an indicator of compromised membrane. This drives the cells toward lipid-deprived conditions, thus disabling cellular homeostasis.

Since the drug treatments disrupted the cellular lipid bilayer (membrane), we further explored the effects of TA on nuclear lipid bilayer (nuclear envelope). The results of TEM studies show distinct changes in nuclear phenotype leading to incapacitation from its functional roles due to drug exposure. These changes in the morphology of the nucleus might be due to the membrane disruption effects of TA on nuclear envelope. Also, similar findings were observed during nuclear fluorescence studies that strengthen our hypothesis of TA’s membrane disruption mechanisms. Subsequently, these incidents severely affect the survival and functional aspects of the cell.

Generally, cancer cells under nutrient-deprived conditions tend to induce ROS, thus promoting the activation of ER stress and UPR^57,58^. Recent studies show phytochemicals induce ROS that mediate cell death by ER stress signaling activation^59,60^. Since TA induced ROS levels in prostate cancer cells, we correlated its mechanism of action in promoting ER stress activation with results observed from iPathwayGuide analysis. Also, we clearly elucidated TA’s role in inducing ER stress-mediated prostate cancer cell death in our previous study^26^. Still, we evidenced the anti-proliferative, anti-migratory, anti-invasive potential of TA from the xCELLigence results. The results of the kinetic study predicted the time-dependent pharmacological effects of TA on prostate cancer cells.

The chemo-sensitizing aspect of TA were assessed through Rhodamine 123 dye accumulation studies. Here, we hypothesized that intracellular dye accumulation mimics the drug accumulations. To substantiate this phenomenon, we have chosen Dox and Doc, therapeutic drugs, and evaluated its efficacy through cell proliferation studies. We found that TA exhibited chemo-sensitivity effects with Dox and Doc treatments. Altogether, we hypothesize that TA promotes ROS-mediated ER stress and inhibit lipid metabolism as well as disrupt cellular membranes. Thus, driving these cells towards apoptosis.

## Conclusion

Our results clearly show that TA has potential anticancer activity against prostate cancer growth through induction of ER stress signaling by ROS production. On other side, TA affected the cellular survival by effectively deregulating the lipid metabolism and disruption of the cellular as well as nuclear membranes. Also, TA was found to be effective in inhibiting the signaling mechanisms that drive the lipogenesis of prostate cancer cells. Moreover, we elucidated the chemo-sensitizing attributes of TA in prostate cancer cells during treatments with standard chemotherapeutic drugs. We therefore suggest TA can be chosen as inhibitor for lipid metabolism and also an adjuvant in combating prostate cancer during standard chemotherapies.

## Materials and Methods

### Cell culture and reagents

Three PCa cell lines C4-2, DU145, and PC-3 (acquired from ATCC, Manassas, VA, USA) were used in this study. The cell lines C4-2, DU145, and PC-3 were cultured in RPMI 1640 (Rosewell park memorial institute medium) complemented with 10% fetal bovine serum and 1 × penicillin–streptomycin antibiotics (Thermofisher, Waltham, USA). Further, these cell lines were grown and maintained at 37 °C in 5% CO_2_.

### xCELLigence studies- proliferation and metastatic assays

To confirm the effects of TA on cellular growth, motility, and invasiveness, we performed new generation real-time cell growth invasion and migration assays by employing the xCELLigence system. This system is a newfangled approach that measures the electrical impedance caused by the growth, migration and invasion of the cells through real time monitoring^61,62^. Concisely, PCa cells (C4-2, PC-3) were seeded in each chamber of the plate for cell proliferation (4 × 10^3^), or in invasion and migration (4 × 10^4^) studies, and the cells were treated with TA at 10 and 20 µM were evaluated for growth inhibition profiles through xCELLigence instrument at 37 °C in 5% CO_2_ through *in vitro* real time monitoring approach.

### Proliferation kinetics

To demonstrate the TA’s cytotoxicity, a cell proliferation kinetic method was followed. In this study, C4-2, DU145, and PC-3 cells of 10,000 cells/well were inoculated in 6 distinct six-well plates (with 1 ml in each well). In the next day, these cells were exposed to 10, 20, and 30 µM TA. Every day, cells were subjected to trypsinization and percent viability was calculated through trypan blue exclusion method by using a Countess Automated Cell Counter^63^ (Invitrogen, Carlsbad, CA, USA). Cell viability was determined by % treated versus non-treated cells multiplied by 100^64^. Since we observed similar inhibitory patterns in C4-2, DU145, and PC-3 cell lines during TA treatments, we have selected C4-2 and PC-3 cells for the subsequent experiments.

### RNA extraction, purification, transcriptome studies, and iPathwayGuide analysis

The RNA was extracted by employing RNA Isolation Kit (Qiagen, Inc., Hilden, Germany), and subsequently, RNA quality was measured by NanoDrop 2000 (Thermo Fisher Scientific, Waltham, MA, USA) and processed for purification through ethanol precipitation as described previously^26,65^. The purified mRNA samples were subjected to gene microarray studies by utilizing Clariom S Human gene array (Affymterix, Santa Clara, CA, USA) per manufacturing instructions. The results of the microarray studies were validated and analyzed for variation in pathways of through iPathway guide analysis (Advaita Corporation, Plymouth, MI, USA)^66^. This method scores the pathway by the level of protein expression in cells during drug treatments through Impact Analysis method^67,68^.

### Determination of intracellular reactive oxygen species (ROS)

Cells after TA exposure were stained with CellROX Deep Red oxidative stress reagent (5 μM; Life Technologies, Carlsbad, CA, USA) and subjected for run on an Accuri C6 Flow Cytometer (BD Biosciences, San Jose, CA, USA). The obtained data was interpreted through using BD accuri C6 software (BD Biosciences). More than 10000 mononuclear cells were chosen in each run within the gated channel drawn in forward and side scatter plot and analyzed for mean fluorescence intensity. The extent of ROS production was ascertained by staining of cells with CellROX Deep Red oxidative stress reagent (5 μM; Life Technologies, Carlsbad, CA, USA)^69^. An Accuri C6 Flow Cytometer (BD Biosciences, San Jose, CA) was employed to evaluate the ROS fluorescence levels.

Also, cells were seeded 1 × 10^4^ in 4-chambered slides (Sarstedt Inc., Newton, NC, USA) and treated using 10 and 20 µM TA. Post 24 h exposure, treated cells were stained with CellROX Deep Red reagent and fixed. Further, cells were permeabilized and nuclei counterstained using a DAPI stain, mounted and imaged using confocal laser scanning microscopy (Carl Zeiss LSM 710, Oberkochen, Germany) at 40X magnification^63^.

### Lipid extraction and FT-IR analysis

Cells (5 × 10^4^ cells/well) at density were inoculated in 6-well plates and grown along with the culture medium. At 70% confluency, cells were exposed to 10 and 20 µM TA for 24 h and incubated. After this, incubation cells were detached, pelleted at speed of 1000 rpm for 5 min.

Total lipid extracts from cell pellets were extracted through Bligh and Dyer method^70^. In brief chloroform (250 µl) and methanol (500 µl) were added to the cell pellets vortexed. Further, 6 M hydrochloric acid (16.8 µl) and additional chloroform (250 µl) were added and vortexed. To this mixture, ultra-pure deionized water (250 µl) was added to this mixture and vortexed again. This mixture was incubated at 4 °C for 1 h and centrifuged at 300 g RCF for five minutes, to allow the segregation of the two phases. The whole lipid extracts were at last collected in the bottom phase.

The lipid composition of C4-2 and PC-3 cells after TA exposure was evaluated through Fourier Transform Infrared (FTIR) (Perkin Elmer Series Spectra 100, Waltham, MA, USA) to validate its molecular effects on cellular lipid profile^71^.

### Western blotting analysis

Cells (1 × 10^6^ cells per plate) were inoculated in 100 mm polystyrene plates and grown. At 70% confluence, cells were exposed to 10 and 20 µM TA for 24 h along with the untreated control. The cells (both dead and live) were pelleted, lysed, and processed for protein profiling studies^72–77^. After TA treatment, cell lysates were prepared using CelLytic M lysis buffer (sigma Aldrich, St. Louis, MO, USA). The whole cell lysates were subjected to SDS-PAGE and transfer process. The transferred membranes were probed with a rabbit anti- AceCS1 (D19C6) (#3658), anti- Phospho-Acetyl-CoA Carboxylase (Ser79) (D7D11) (#11818), anti- Acetyl-CoA Carboxylase (C83B10) (#3676), anti ATP-Citrate Lyase (#4332), anti- Phospho-ATP-Citrate Lyase (Ser455) (#4331), and anti-Lipin 1 (D2W9G) (#14906), anti-ACSL1 (D2H5) (#9189) (cell signaling, Danvers, MA, USA) trailed by a peroxidase-conjugated, anti-rabbit IgG antibody (cell signaling, Danvers, MA). Protein expression were then developed using ChemiDoc MP Imaging System (Bio-Rad, Hercules, CA, USA)^78^.

### Plasma membrane staining

Cells (1.0 × 10^4^/well) were seeded on a 4-chambered slide and grown till 24 h and then treated for 24 h with 10 and 20 μM TA. After 24 h incubation, cells were then subjected to CellMask Deep Red plasma membrane stain with pseudo color red. Additionally, the cells were fixed through 2% paraformaldehyde for 25 min then 1X PBS washed thrice and permeabilized using 0.2% triton X100. Finally, cells were counter stained using DAPI and processed with mounting medium. The cells were examined through an inverted confocal spectral laser scanning system (Carl Zeiss AG, Oberkochen, Germany), and images were acquired and analyzed through ZEN software^79^.

### Membrane integrity assay

To determine plasma membrane integrity during TA treatment, we performed membrane integrity by employing the CytoTox-ONE Homogenous Membrane Integrity Assay (Promega, Madison, WI, USA), that quantifies lactate dehydrogenase (LDH) release which was characteristic attribute of cell during death. Cells were plated at a density of 5.0 × 10^3^ cells/well in luminometric 96-well plates, grown till 24 h, and then treated for 24 h with 10, 20 and 30 μM TA. The measure of cell death was demonstrated and performed through a LDH-based CytoTox-ONE Homogeneous Membrane Integrity Assay (Promega, Madison, WI) based on the manufacturer’s protocol^80^. The quantification of LDH release directly corelates with the amount of cell survival. The assessment of fluorescence in the sample was measured at emission spectra at 590 nm with excitation set at 560 nm using Cytation5 microplate reader (BioTek Instruments, Inc., Winooski, VT, USA).

### Zeta potential

To evaluate lipid integrity of the plasma membrane after the TA treatment, we measured the cell’s surface charge through Zeta potential analysis. Cells (2.5 × 10^5^/well) were seeded on a 6-well plate and grown till twenty-four h and then treated for 24 h with 10, 20, and 30 μM TA. Next, cells were trypsinized and centrifuged. The resulting cell pellet of 10 μl volume was added to 1 ml of deionized water and Zeta potential (surface charge) measured instantly through Zetasizer (Nano ZS, Malvern Instruments, Malvern, UK) based on dynamic light-scattering principle technique^81^.

### Nuclear morphological studies

To further validate impact of TA on nuclear morphology, transmission electron microscopy (TEM) study was performed. This study offers apparent evidence of the effect of TA on the nucleus at higher magnification. For this experiment, cells (1 × 10^7^/150 mm plate) were grown and treated with TA (10 μM) for 24 h. These cells were pelleted, fixed using standard ice-cold formaldehyde (4%)-glutaraldehyde (1%) fixative solution trailed by osmium tetroxide exposure, and imaged under TEM^82^.

### DAPI staining

Cells (1 × 10^4^) were seeded on a 4-chambered slide in each well and grown till 24 h and then exposed for 24 h with 10 and 20 μM TA. Treated cells were fixed with 2% paraformaldehyde for 25 min, rinsed with 1X PBS thrice for 5 min each, and then permeabilized with 0.2% Triton X-100 for 5 min. Then, cells were subjected to 1X PBS wash, twice for 5 min each. The slides were stained and processed using DAPI containing mounting medium^83^. Finally, cells were imaged through confocal laser scanning microscopy (Carl Zeiss LSM 710, Oberkochen, Germany)^63^ using 40X oil immersion objective.

### Rhodamine 123 uptake/retention assays

Cells (2.5 × 10^5^) were plated in 6-well tissue culture plates (Costar, Cambridge, MA, USA) for the retention/uptake of Rh123 dye. Further, cells were treated with 10, 20 and 30 μM of TA for 24 and dye uptake was conducted by addition of Rh123 (200 ng/ml) in each well for 1h. Then, cells were rinsed and added with culture medium containing no Rh123 and incubated at 37 °C for 90 min. Through the measure mean fluorescence intensity values from the histogram data and the efflux of Rh123 from the intracellular region was evaluated. Each sample were run in triplicates and in every run 10000 events were captured within the gate drawn. The fluorescence was acquired at emission wavelength 525 nm^84^.

### MTS Cell proliferation studies

Cells were (5 × 10^3^ cells/well) plated at a concentration of 5 × 10^3^ cells/100 μl in 96-well plates and grown in culture incubator (Series 8000 Direct-Heat CO_2_, Thermofisher, Waltham, MA, USA) for 24 h. The cells were then exposed to (0.625, 1.25, and 2.5 µM) concentrations of Dox and (2.5, 5.0, and 10 nM) concentrations of Doc in the presence of TA (10 and 20 µM). The PBS was used as control. Post 24 h drug exposure, the cells were rinsed with 1X PBS and added with MTS reagent for 2 h. The absorbance maxima were acquired at 490 nm by utilizing the microplate reader (Cytation 5 imaging reader, BioTeK, Winooski, VT, USA). The percent viable cells were calculated as previously reported method^85–89^.

### Statistical analysis

The results are articulated as the standard error mean. The means were compared to those of untreated control cells by Student’s t-test using GraphPad Prism 5 software program (purchased from the GraphPad Software Inc. La Jolla, CA, USA). All statistical analysis was achieved using student’s t test. P value of <0.05, 0.01, and 0.001 were measured to represent statistically significant difference w.r.t controls.

## Supplementary information

Supplementary Information
