# Supplementary material for: Tannic acid inhibits lipid metabolism and induce ROS in prostate cancer cells
Source: Sci Rep. 2020 Jan 22;10:980. doi: 10.1038/s41598-020-57932-9 (PMC6976712; doi:10.1038/s41598-020-57932-9)
Supplement: Supplementary file 1 — Supplementary Information [file 41598_2020_57932_MOESM1_ESM.docx]

**Tannic acid inhibits lipid metabolism and induce ROS in prostate cancer cells**

Prashanth K.B. Nagesh^1,2^, Pallabita Chowdhury^2^, Elham Hatami^2^, Shashi Jain^3,4^, Nirnoy Dan^2^, Vivek Kumar Kashyap^1,2^, Subhash C. Chauhan^1,2^, Meena Jaggi^1,2^, Murali M. Yallapu^1,2*^

^1^Department of Microbiology and Immunology, School of Medicine, University of Texas Rio Grande Valley, McAllen, TX 78504, USA

^2^Department of Pharmaceutical Sciences and Center for Cancer Research, University of Tennessee Health Science Center, Memphis, TN 38163, USA

^3^Tumor Initiation and Maintenance, Sanford-Burnham Medical Research Institute, La Jolla, California 92037, USA.

^4^Department of Pathology, Moores UCSD Cancer Center, and Sanford Consortium for Regenerative Medicine, University of California, San Diego, La Jolla, CA 92037, USA.

**Funding Sources**

This work was supported by the National Institute of Health/National Cancer Institute’s funding: K22 CA174841 awarded to MMY. The studies were also grateful for partial support from the National Institute of Health grants R01 CA210192, R01 CA206069, and R01 CA204552 awarded to SCC.

***Corresponding Author:**

Murali M. Yallapu, Ph.D.

Associate Professor

Department of Immunology and Microbiology

5300 North L Street, Room 2.249

McAllen, TX 78504

Phone: (956) 296-1705

Fax No: (956)-296-1325

E-mail: [murali.yallapu@utrgv.edu](mailto:murali.yallapu@utrgv.edu)

**Competing interests**

The author(s) declare no competing interests.


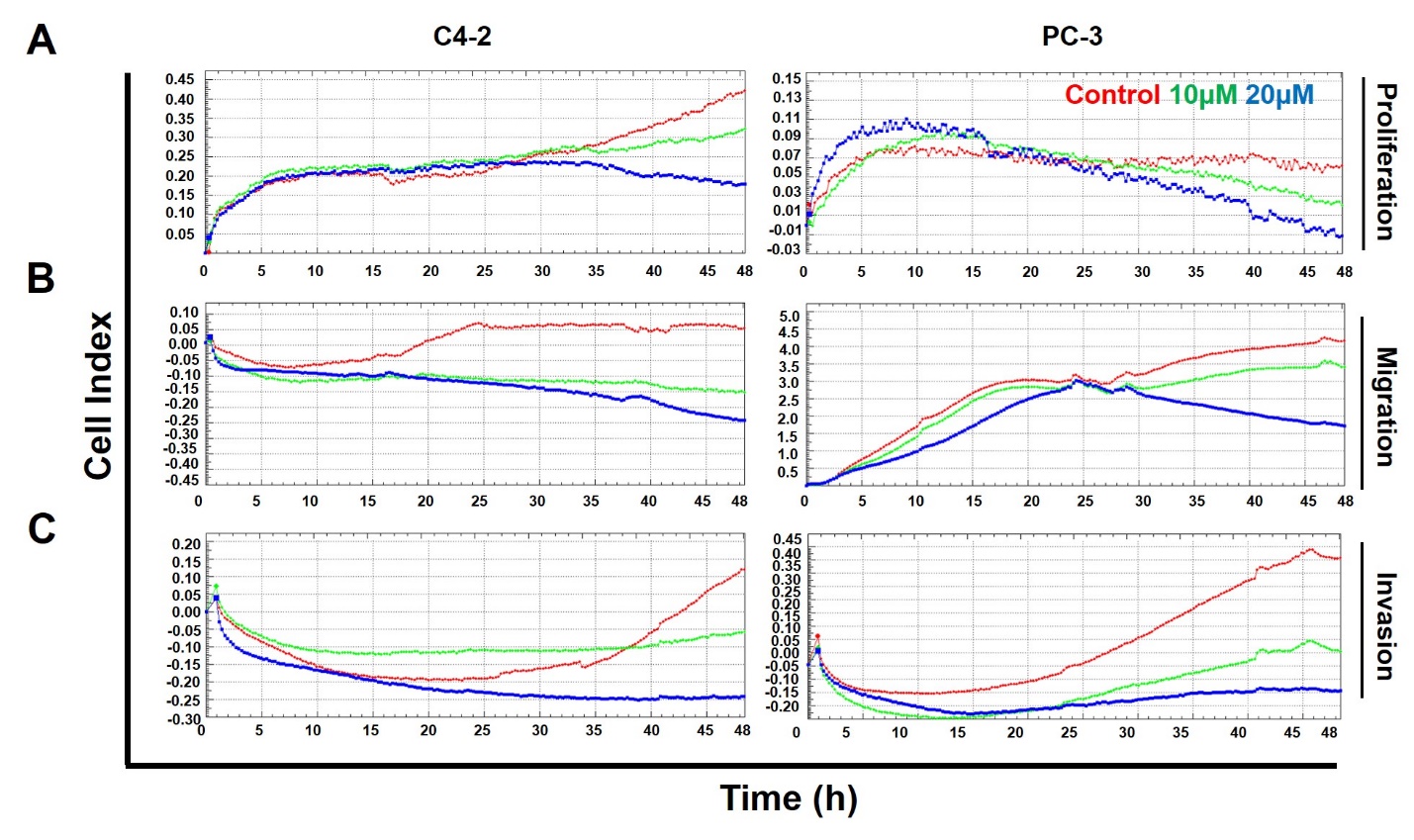


**Supplementary figure 1: Anti-proliferative and anti-metastatic effects of TA. A-C)** xCELLigence system demonstrating superior efficacy of TA on PCa cells as shown by real time cell proliferation, migration, and invasion assays. **A)** Effect of TA on cell proliferation ability of PCa cells with respect to time (h) **B)** Effect of TA on C4-2 and PC-3 cell migration ability **C)** Effect of TA on C4-2 and PC-3 cell invasion ability.

**Full-length bolts of Western studies represented in Figure 5C**


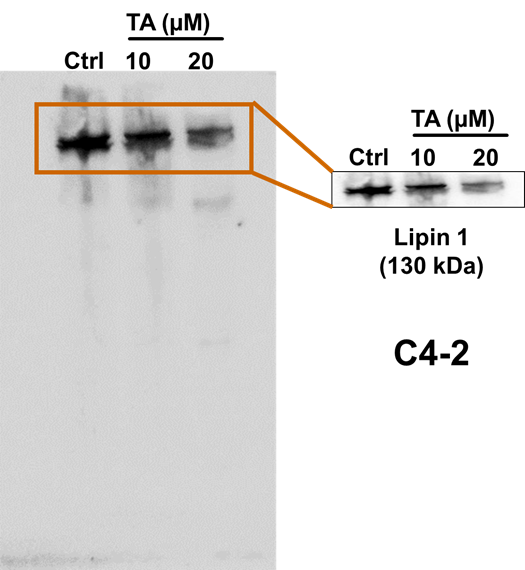


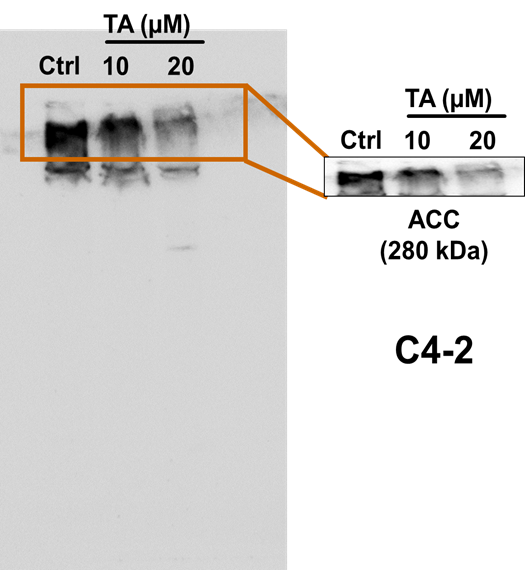


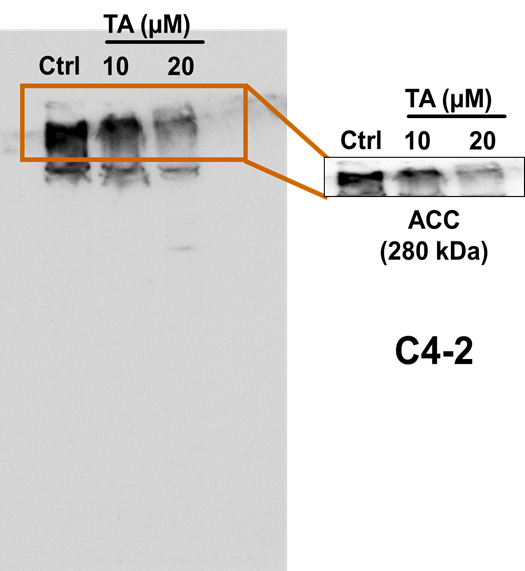


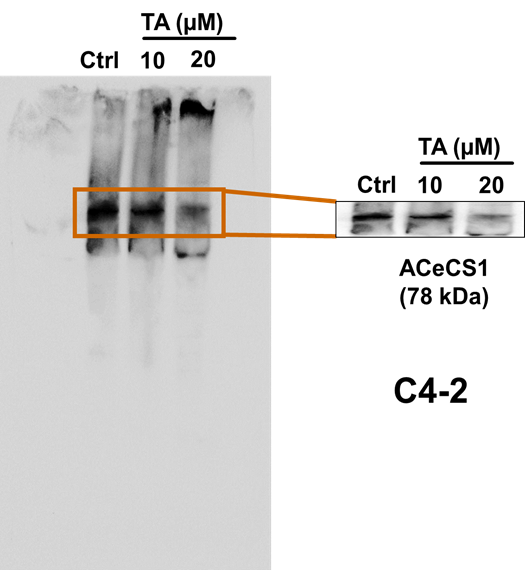


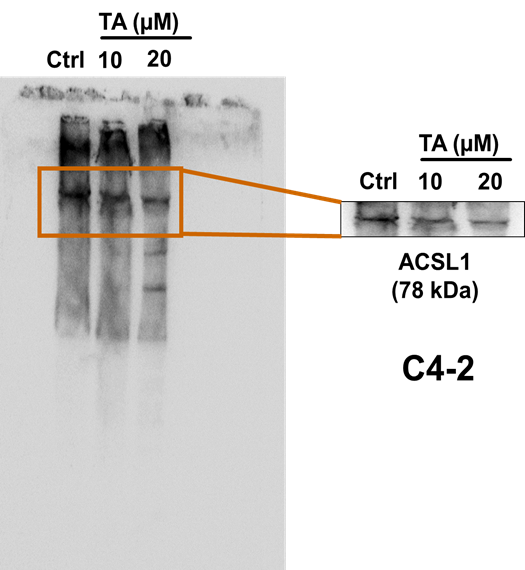


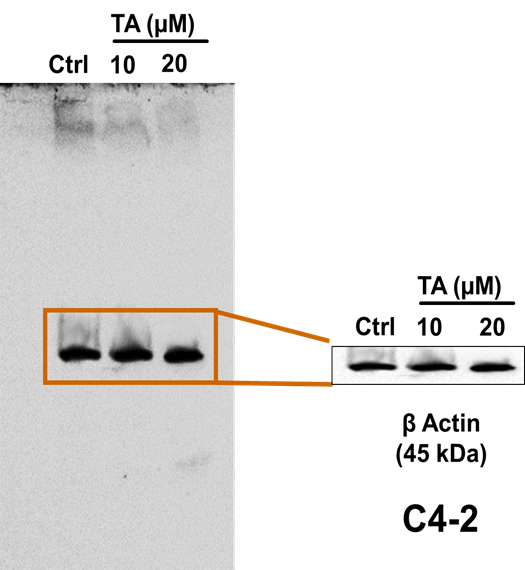


**Supplementary figure 2**: Full western blots for proteins of C4-2 cells shown in figure 5C. The protein ladder used for these bolts’ ranges from 10 kDa to 250 kDa (Bio-rad cat#1610374, Hercules, CA).


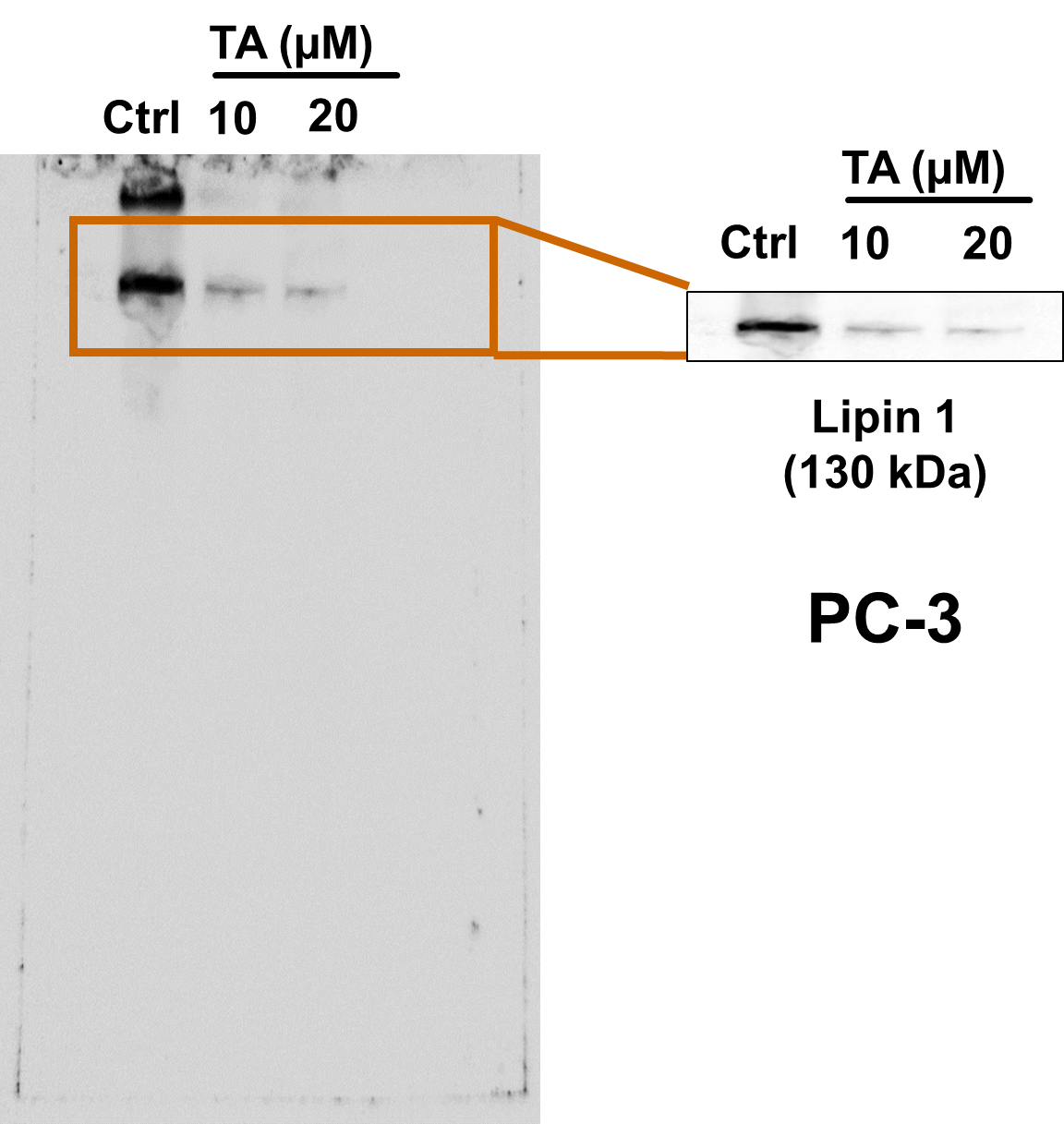


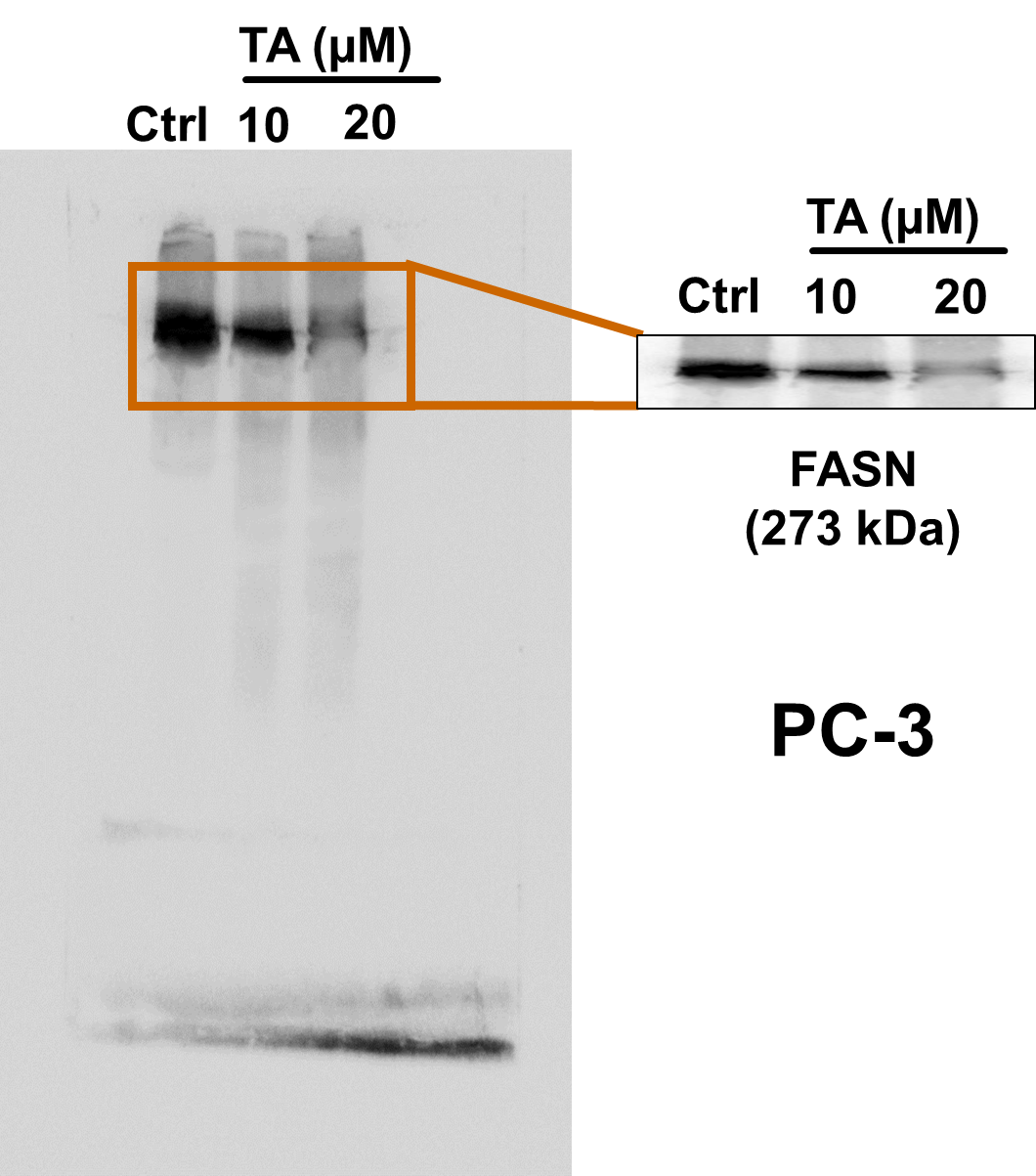


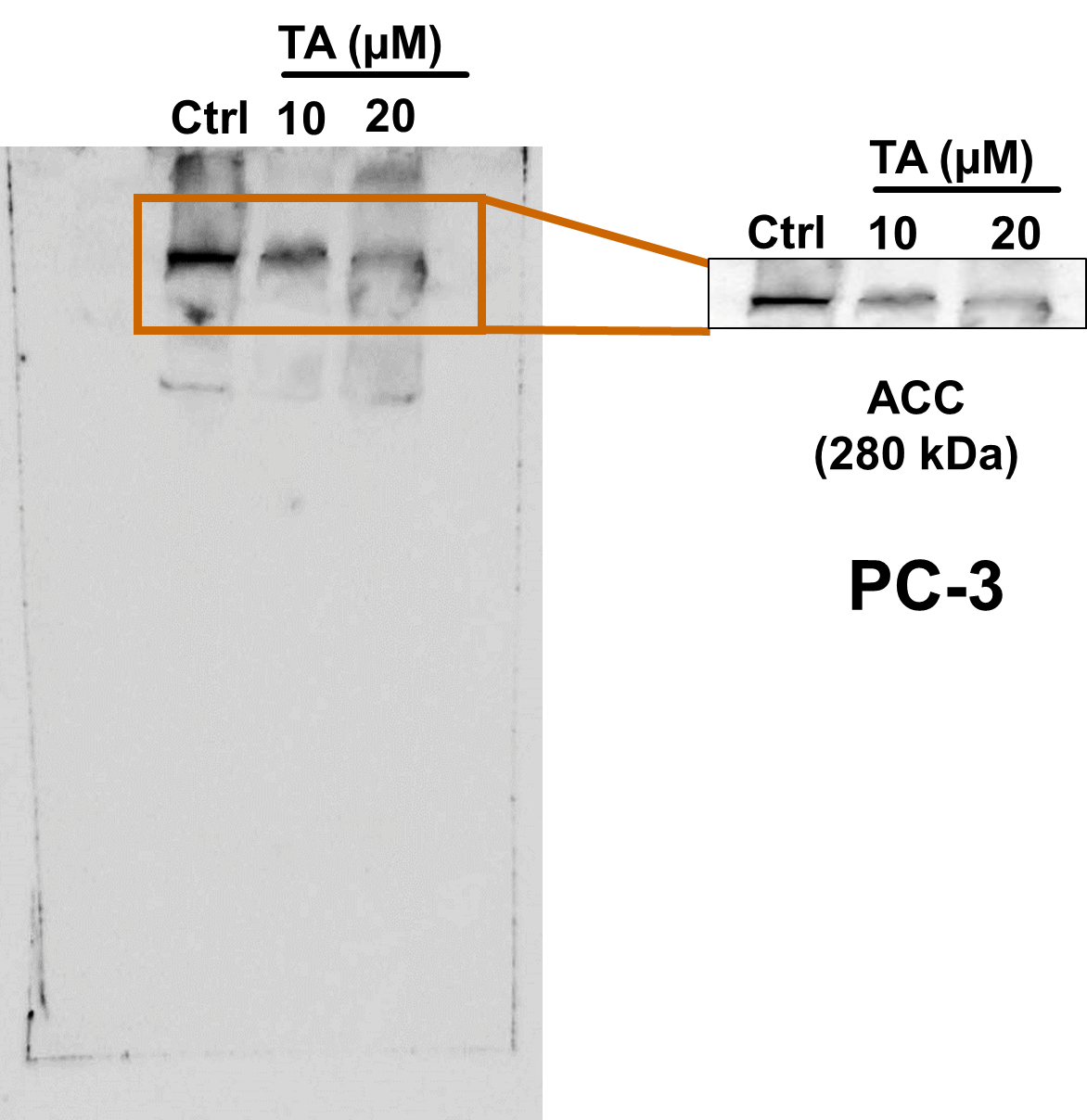

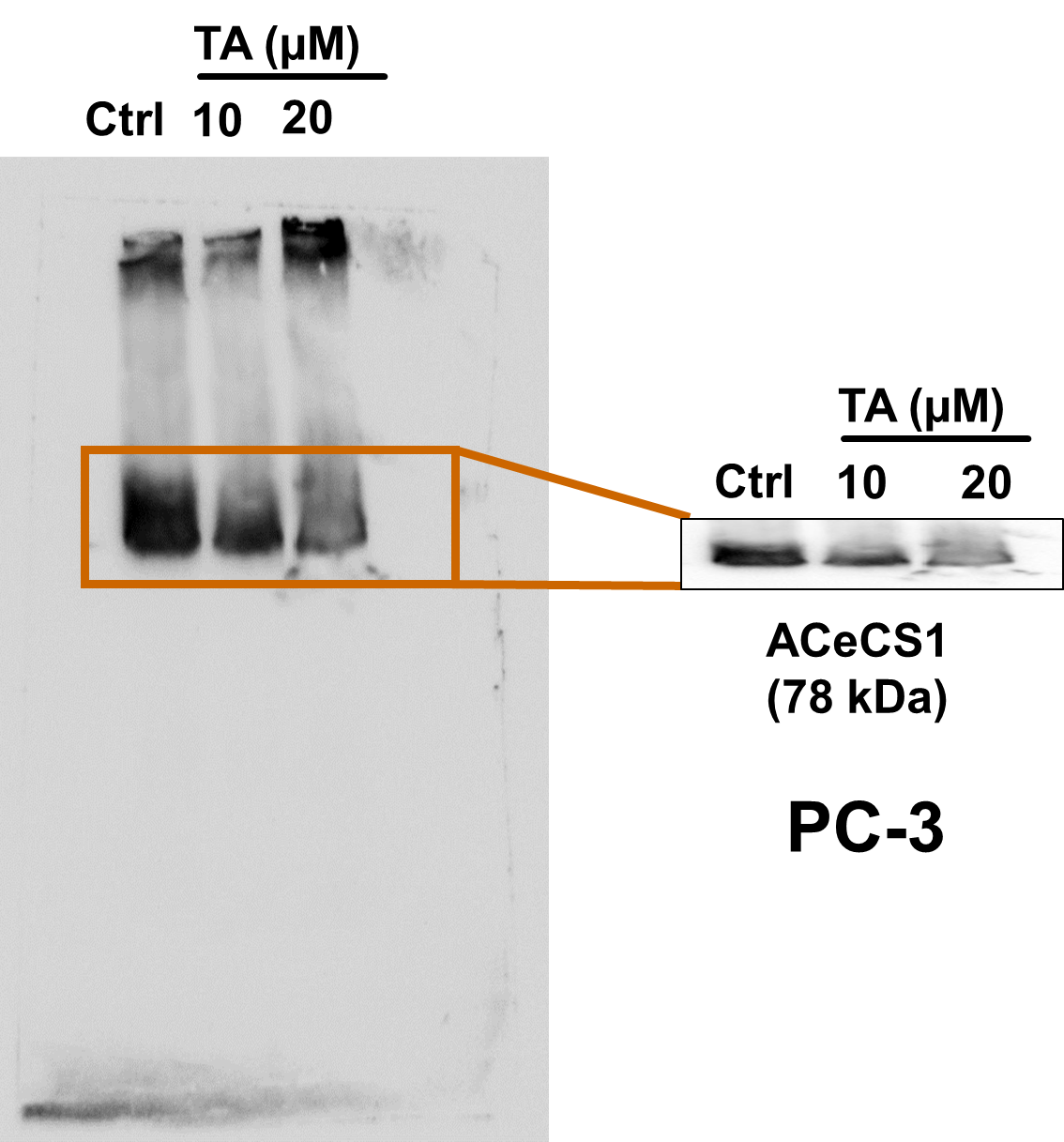

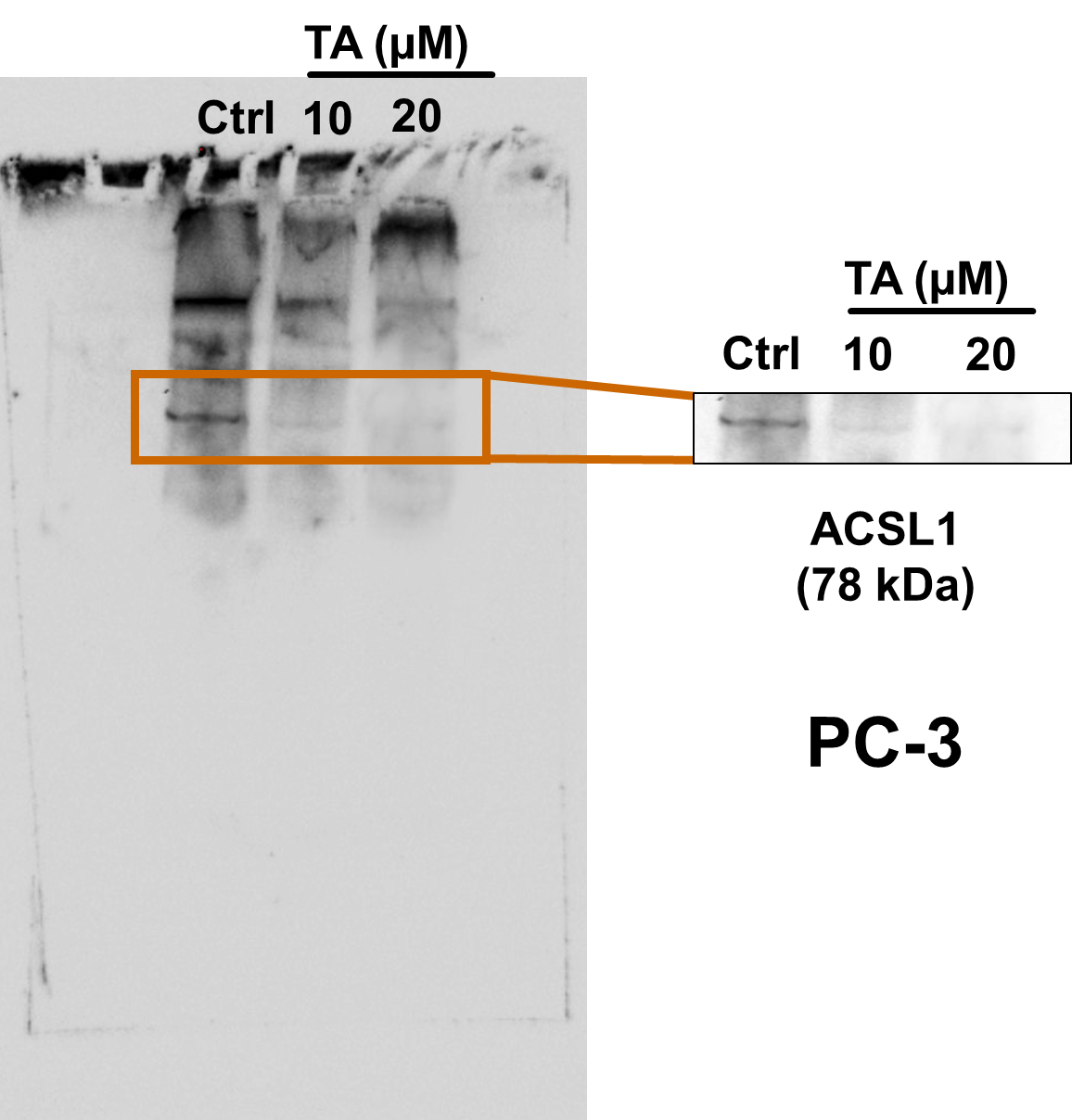


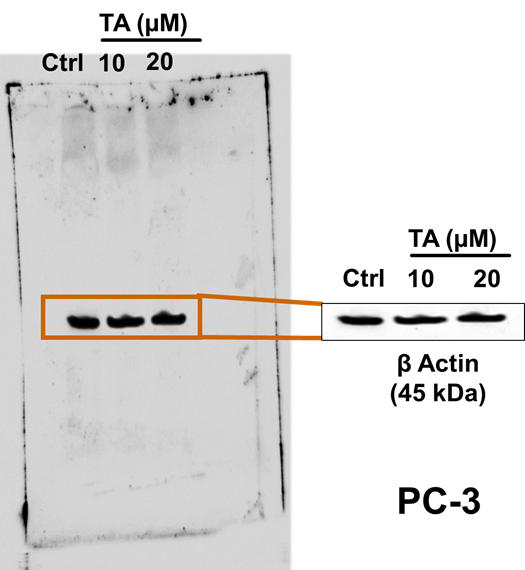


**Supplementary figure 3**: Full western blots for proteins of PC-3 cells shown in figure 5C. The protein ladder used for these bolts’ ranges from 10 kDa to 250 kDa (Bio-rad cat#1610374, Hercules, CA).
